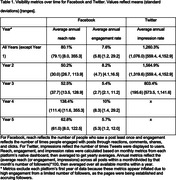# Aging and ADRD social media programs for outreach and recruitment: Longitudinal outcomes

**DOI:** 10.1002/alz.090770

**Published:** 2025-01-09

**Authors:** Anthony L. Teano, Ashley Scott, Cassandra Gipson, Marilyn S. Albert, Corinne Pettigrew

**Affiliations:** ^1^ Johns Hopkins University School of Medicine, Baltimore, MD USA; ^2^ Johns Hopkins University, Baltimore, MD USA

## Abstract

**Background:**

Social media may be a useful method for research centers to deliver health messaging, increase their visibility in the local community, and recruit study participants. There are, however, few studies evaluating the outcomes of social media in this setting. The objective of this study was to describe one Alzheimer’s Disease Research Center’s social media activities for community education on topics related to aging, memory loss, and dementia, and evaluate their impact on recruitment into clinical research studies.

**Method:**

Several social media platforms were used, including: Facebook, Twitter/X, and YouTube. Objective assessments quantified monthly, based on each platform’s native dashboard, included: number of posts, number of followers, post reach and engagement, and post impressions. The number of participants volunteering for research during this period was also tracked using a RedCap database, with many entries reflecting individuals who indicated that they learned about Center research through social media when completing a “Participate in Research” webform. Educational material posted to social media most frequently included content developed by Center staff, content from partner organizations, and news articles/resources featuring Center researchers. Multiple educational programs were developed, including social media series, virtual talks, Twitter chats, and webinars. Facebook content was occasionally boosted to increase visibility in the local geographical region.

**Result:**

Metrics over four years demonstrate growth in reaching social media audiences, as indicated by increases over time in the numbers of likes/followers on Facebook and Twitter and video views on YouTube (growth trajectories). Overall, Facebook reach rates and Twitter impression rates were respectable, although Facebook engagement rates were more modest (Table 1). Months that included boosted Facebook posts resulted in a greater change in page Followers and page Likes, and higher reach and engagement rates (all *p*£.002). Recruitment of participants into Center‐affiliated research studies increased during this time frame, particularly when Facebook posts were boosted.

**Conclusion:**

These data demonstrate that social media activities can provide increased educational opportunities focused on Alzheimer’s disease and related dementias and have a measurable impact on recruitment of participants into research studies. Additionally, this study highlights the importance of tracking outcomes to maximize return on investment.